# Efficient inhibition of HIV-1 expression by LNA modified antisense oligonucleotides and DNAzymes targeted to functionally selected binding sites

**DOI:** 10.1186/1742-4690-4-29

**Published:** 2007-04-26

**Authors:** Martin R Jakobsen, Joost Haasnoot, Jesper Wengel, Ben Berkhout, Jørgen Kjems

**Affiliations:** 1 Department of Molecular Biology, University of Aarhus C.F. Møllers Allé, building 130, DK-8000 Århus C, Denmark; 2 Department of Human Retrovirology Academic Medical Center, University of Amsterdam, Meibergdreef 15, 1105 AZ, Amsterdam, The Netherlands; 3 Department of Chemistry, University of Southern Denmark, Campusvej 55, DK-5230 Odense M, Denmark

## Abstract

**Background:**

A primary concern when targeting HIV-1 RNA by means of antisense related technologies is the accessibility of the targets. Using a library selection approach to define the most accessible sites for 20-mer oligonucleotides annealing within the highly structured 5'-UTR of the HIV-1 genome we have shown that there are at least four optimal targets available.

**Results:**

The biological effect of antisense DNA and LNA oligonucleotides, DNA- and LNAzymes targeted to the four most accessible sites was tested for their abilities to block reverse transcription and dimerization of the HIV-1 RNA template *in vitro*, and to suppress HIV-1 production in cell culture. The neutralization of HIV-1 expression declined in the following order: antisense LNA > LNAzymes > DNAzymes and antisense DNA. The LNA modifications strongly enhanced the *in vivo *inhibitory activity of all the antisense constructs and some of the DNAzymes. Notably, two of the LNA modified antisense oligonucleotides inhibited HIV-1 production in cell culture very efficiently at concentration as low as 4 nM.

**Conclusion:**

LNAs targeted to experimentally selected binding sites can function as very potent inhibitors of HIV-1 expression in cell culture and may potentially be developed as antiviral drug in patients.

## Background

Targeting specific mRNAs by annealing complementary oligonucleotides is a basic principle of several different gene silencing technologies. In the simplest form, antisense single stranded oligonucleotides (or derivatives hereof) are introduced into the cell to block gene expression by interfering with translation of the mRNA or by degrading the RNA in a DNA/RNA heteroduplex via an RNaseH dependent pathway. This antisense approach has been used for more than two decades to study gene function in the laboratory and in attempts to treat animal and human diseases [1-4]. However, the antisense technology has never fulfilled the initially anticipated break-through as a therapeutic tool. Poor intracellular delivery, *in vivo *instability of the single stranded oligonucleotide, chemical toxicity and lack of mRNA target accessibility are possibly obstacles for a lacking antisense effect. The latter problem is mainly due to the formation of stable RNA structures and assembly of the mRNA into nucleoprotein complexes rendering the target site inaccessible to base pairing [5,6]. Furthermore, it has been estimated that only 2–5% of randomly chosen antisense oligonucleotides have any effect on gene expression [5,7] and computer generated structure models are generally not sufficient for rational prediction of effective targets.

In a related approach, RNA- or DNA-based endonucleases (ribozymes and DNAzymes) are used to cleave complementary targets in mRNA. The most commonly used ribozyme, the hammerhead, has been used extensively in vitro and with more limited success in vivo (reviewed in [8,9]). One of the main reasons is probably the notorious instability of unmodified RNA in vivo. DNAzymes do not appear to exist in nature, but have been selected *in vitro *from random DNA oligo pools. One of the most active DNAzymes, named 10–23, bears some structural resemblance to the hammerhead ribozyme [10-12] but, in spite of the higher in vivo stability of single stranded DNA compared to RNA, it also demonstrated only variable success *in vivo *[13]. In the reported examples of targeting nucleic acid enzymes to HIV-1 RNA, either relatively large concentrations and combination of catalytic molecules are required or an in vivo expression system is used [14-17]. Common to both the antisense and the enzymatic approach are that the knock down efficacy is restricted by the accessibility of the targets in the mRNA *in vivo*.

More recently, RNA interfering (RNAi) has been developed as a highly potent approach to knock down gene expression in mammalian cells with an unprecedented efficiency and specificity (Reviewed in [3]). The active molecule is a small interfering RNA (siRNA), a 20–23 nucleotides RNA duplex composed of two complementary strands, one of which is complementary to the mRNA target. Although it was initially suggested that the siRNA approach is less sensitive to RNA structure in the target, it was recently demonstrated that the efficiency of RNAi-mediated "knock down" can also be influenced by the RNA structure in HIV-1 [18-21].

**Figure 1:**
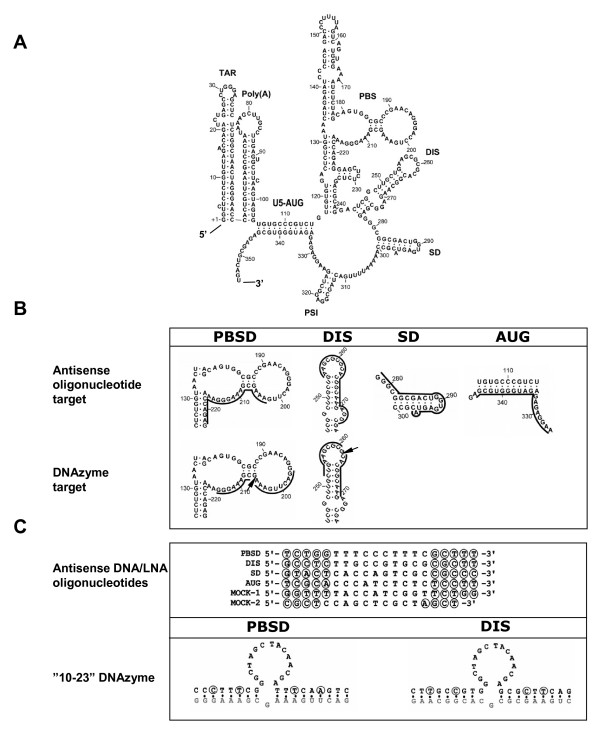
Oligonucleotides and their respective targets in the 5' end of the HIV-1 RNA genome. (A) Secondary structural model of the HIV-1 leader RNA. The stem-loops are named according to assigned function (see text for details) and the sequence is numbered from the 5' end of the RNA transcript. (B) The targets for the various oligonucleotide constructs. The annealing sites for the oligonucleotides are indicated by a solid line and the cleavage sites of the DNA/LNAzymes are marked by arrows. (C) Sequences of the antisense oligonucleotides and DNAzymes containing the 10–23 catalytic motif [10] named according to their target sites shown in panel B. The selected target sequences for the antisense constructs include sequences downstream of the primer binding site (PBSD), the dimerization initiation site (DIS), the splice donor site (SD) and the Gag initiation codon (AUG). The nucleotides that are substituted with LNA residues in the LNA antisense gap-mers and LNAzymes constructs are circled. The target sequences of the "10–23" DNAzymes are indicated with grey letters.

To address the general problem of accessibility of mRNA we have previously developed a SELEX approach that selects for the most effectively binders from a 20-mer complete antisense library through repeated binding cycles [22]. The selection protocol was applied specifically to the 355-nucleotides 5'-terminal fragment of the HIV-1 RNA genome because: a) it contains several functionally important elements including the *trans*-activation response element (TAR), the 5' polyadenylation signal (Poly(A)), the primer binding site (PBS), the dimer initiation site (DIS), the major splice donor (SD) and the packaging signal (PSI) that precedes the Gag open reading frame (Fig. 1A; reviewed in [23,24]); b) most of the region is positioned upstream of the major splice donor site and is therefore present in all viral mRNA species; c) this region is scanned by the ribosome prior to cap-dependent protein synthesis; d) it is the most conserved region of the HIV-1 genome, thus increasing the chance that all HIV-1 strains are inhibited and reducing the likelihood of escape mutants; and e) we have previously tested several siRNA targeted to this region and in all cases non or very low efficiencies were observed (unpublished data). This study revealed four sites that are particularly accessible to antisense binding and these targets are here subjected to further analysis.

Chemical modifications are often introduced at the ribose and/or phosphate group of the backbone to increase the stability of oligonucleotides for *in vivo *applications. In this report the antisense effect of DNA and DNAzyme was compared to oligos that are modified with locked nucleic acid (LNA) residues. This modification consist of a methylene bridge that connects the 2'oxygen with the 4'carbon of the furanose ring, This modification locks the structure into the C3'-endo configuration, which is ideal for recognition of RNA motifs, renders the nucleic acid inaccessible the nucleases and increases the melting temperature with the RNA target strands by 2–7°C per LNA residue [25,26]. To enable efficient RNaseH cleavage of the target mRNA by the antisense oligo, it is important to avoid LNA residues in a stretch of at least 6 nucleotides, a design generally referred to as a gap-mer [27-29]. Moreover, in the design of DNAzymes with LNA modifications it has been reported that 2–3 modifications in each arm gives the optimal binding affinity versus binding kinetic [30,31]. Here we tested DNA and LNA (gap-mer) antisense oligos, DNA- and LNAzymes directed towards four highly accessible targets in the HIV-1 leader. We found that the LNA antisense is the most potent inhibitor, neutralizing viral expression efficiently when applied in nanomolar concentrations. The LNAzymes had a moderate effect, whereas unmodified DNA/DNAzymes have no or very little effect.

## Results

### Construct design and LNA modification of targeting oligonucleotides

Four target sites were selected in the HIV-1 5'-UTR as potential target based on previous accessibility selection studies [22]: 1) a region immediately downstream from the primer binding site (PBSD, 203–222), 2) a region covering the DIS (DIS, 255–274), 3) a region encompassing the major splice donor site (SD, 278–297) and 4) a region covering the gag initiation site (AUG, 326–345; Fig. 1). Four different types of oligonucleotides with potential interfering properties were synthesized: DNA antisense, LNA antisense, "10–23" DNAzyme and "10–23" LNAzyme. All DNA- and LNA-antisense constructs contained 20 nucleotides that were complementary to the selected targets in the HIV-1 RNA, whereas the DNA- and LNAzymes contained two arms of 8–9 nucleotides complementary to the target (Fig. 1B and 1C). The LNA antisense oligonucleotides were designed as gap-mers with 5 LNA residues flanking a 10-mer phosphorothioate modified DNA body to enable RNase H cleavage. The incorporation of the LNA monomers was calculated to raise the T_m _values by approximately 20 degrees.

### Blocking reverse transcription with LNA oligonucleotides

**Figure 2:**
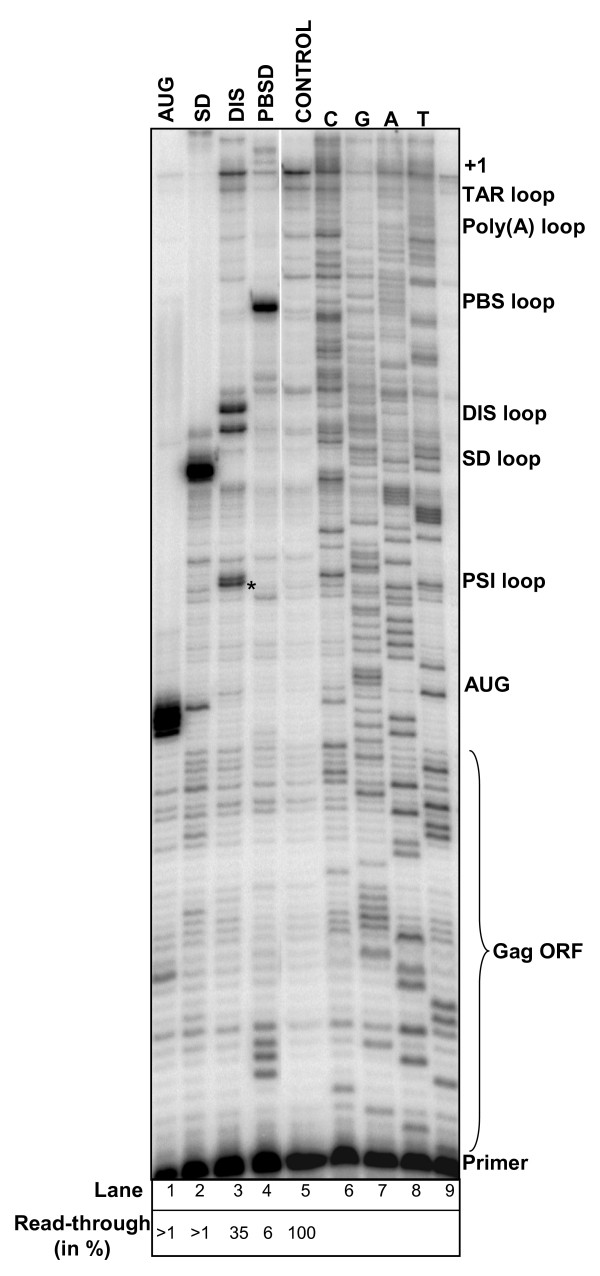
The effect of antisense LNA on reverse transcription of HIV-1 RNA. An equimolar amount (1pmol) of HIV RNA and LNA oligo was mixed and incubated prior to primer extension using a primer complementary to position 384–401. Antisense LNA oligonucleotides, included LNA_AUG _(lane 1), LNA_SD _(lane 2), LNA_DIS _(lane 3), LNA_PBSD _(lane 4), and the major sites of transcriptional termination are indicated to the right. Read through to the 5' end of the HIV-1 RNA is denoted by +1. A sequence latter obtained by dideoxysequencing of the HIV-1 RNA is included in lanes 6–9. The level of read through reverse transcription is calculated as read-through/(read through + paused) × 100% and indicated below.

**Figure 3:**
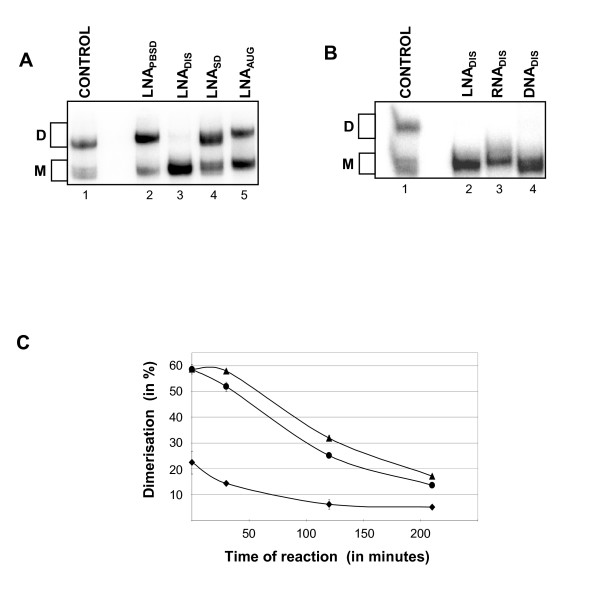
The effect of antisense LNA versus RNA and DNA on dimerization of HIV-1 RNA. The ability of (A) antisense LNA oligonucleotides directed towards different targets or (B) antisense LNA, RNA or DNA oligonucleotide directed towards the DIS target, to inhibit the formation of the DIS dimer-complex during 30 min incubation were investigated. Monomeric and dimer bands are indicated to the left. (C) A similar experiment, but where the indicated antisense oligonucleotide was added after the dimers were allowed to pre-form for 30 mins and subsequently incubated for the indicated time, hence evaluating the efficiency of breaking a stable DIS dimer-complex as a function of time time. Diamonds = LNA_DIS_; Bullet = RNA_DIS_; Triangle = DNA_DIS_.

Reverse transcription of the RNA genome into DNA is an essential step in the viral replication cycle, and antisense oligonucleotides may inhibit this step. We therefore tested the ability of the four LNA antisense oligonucleotides to inhibit reverse transcription in vitro (Fig. 2). The PBSD, SD, and AUG specific LNAs blocked reverse transcription from a downstream primer almost completely and precisely at the expected site (94–99%; Fig. 2, lanes 1, 2 and 4), whereas the LNA_DIS _only showed a partial effect (Fig. 2, lane 3). Interestingly, the latter effect is not caused by insufficient binding of the LNA to DIS, since this LNA inhibits RNA dimerization almost completely (Fig. 3A). The extra band observed at a position corresponding to the PSI hairpin when adding LNA_DIS _can be explained by partial sequence complementarity between the LNA and this region (Fig. 2 lane 3, marked by asterisk). When using the DNA versions of the same oligonucleotides only 60–70% inhibition of reverse transcription was observed for any of the selected sites ([22]; data not shown), clearly demonstrating the superior stability of RNA-LNA duplexes.

Of all the LNAs tested only LNA_DIS _blocked HIV-1 RNA dimerization and with an efficacy of 97% if added to the dimerization reaction prior to incubation (Fig. 3A). Similar levels of inhibition were observed for DNA and RNA oligos (Fig. 3B). However, if the antisense oligonucleotides were added after pre-dimerization of the HIV-1 RNA, the LNA modified antisense oligonucleotide was significantly more potent then RNA and DNA in dissociating the dimer (Fig. 3C).

### Enzymatic cleavage of the HIV-1 leader sequence with DNAzymes and modified LNAzymes optimized for binding

**Figure 4:**
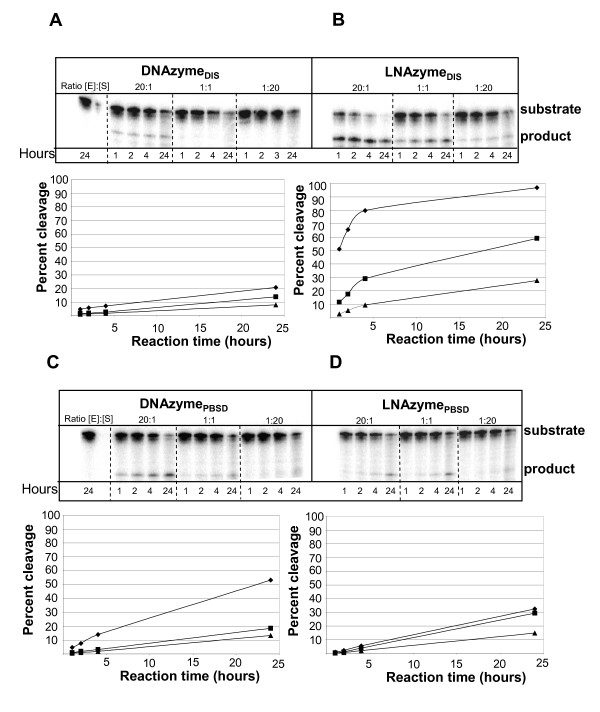
*In vitro *cleavage of HIV-1 RNA by DNA- and LNAzymes. One hundred nmol 5' end labeled leader RNA (+1–355) was incubated with 5 nmol, 100 nmol or 2 pmol DNAzymes or LNAzymes for the indicated time. The DNAzymes targeted to the PBSD and DIS regions cleaved primarily at the expected site, yielding a 5'-end labeled fragment of 205 and 261 nucleotides, respectively (product; panel A and C). The same bands were obtained using the LNAzyme (panel B and C). The experiment was made in duplicates yielding essentially the same result and the cleavage efficiencies indicated below each autoradiogram were calculated as (cleaved RNA/cleaved RNA and uncleaved RNA) × 100% averaged over both experiments.

We wanted to investigate whether the selected regions in the HIV-1 leader were accessible to enzymatic cleavage by DNAzymes. Nucleotide enzymes targeting the selected DIS and PBS sites were synthesized both as DNA (DNAzyme_DIS _and DNAzyme_PBSD_, respectively; Fig. 1) and with two LNA modifications in each arm (LNAzyme_DIS _and LNAzyme_PBSD_, respectively). The cleavage efficiency was assessed by incubating 5'-end radioactively labeled HIV-1 leader RNA with the DNAzymes or LNAzymes for different time points at 10 mM Mg^2+ ^at an enzyme to substrate ratio of 20:1, 1:1 to 1:20. The HIV RNA cleavage products were separated by denaturing gel electrophoresis and quantified (Fig. 4). The DNAzyme_DIS _and DNAzyme_PBSD _and their LNA modified counterpart oligonucleotides cleaved the HIV-1 RNA at the expected position, producing 5'-end labelled fragments of approximately 261 and 205 nucleotides, respectively.

When incubating the HIV-1 RNA with an excess of enzyme (20:1) both DNAzymes showed significant levels of cleavage after 24 hours (Fig. 4A and 4C). At lower stoichiometric amounts (1:1 and 1:20) only DNAzyme_PBSD _showed moderate cleavage in the PBS loop after 24 hours (Fig. 4C). Introduction of LNA in the arms of DNAzyme_DIS _strongly induced the efficacy to nearly 100% cleavage after 24 hours (20:1 excess) and to a moderate cleavage level at lower enzyme concentration (1:1 and 1:20) (Fig. 4B). In contrast, LNA modifications did not improve the activity of DNAzyme_PBSD _(Fig. 4D). A small decline in its inhibitory activity was measured, indicating that the advantage of introducing LNA residues into a DNAzyme is not universal but rather depends on the nature of the target.

### Blocking expression of HIV-1 *in vivo*

**Figure 5:**
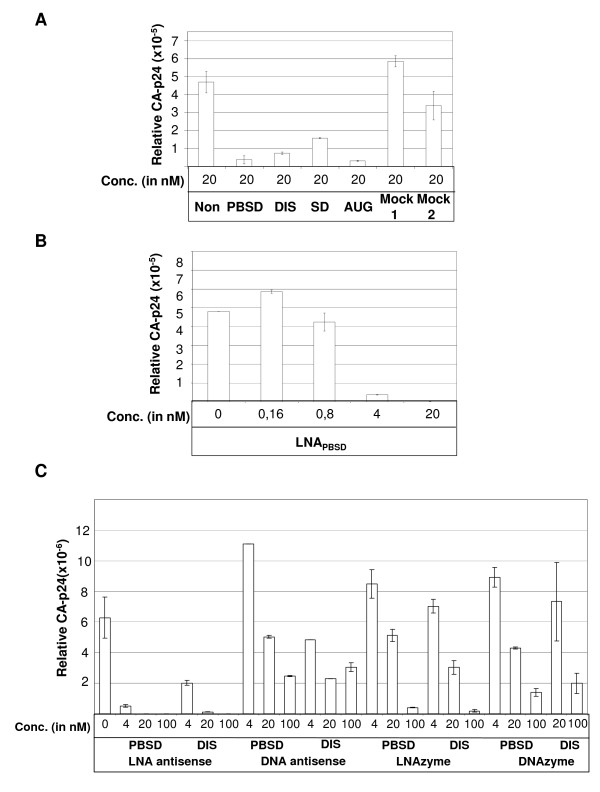
Inhibition of intracellular HIV-1 production in the presence of antisense LNA and LNAzymes. One hundred nanograms of HIV-1 genomic LAI plasmid was co-transfected with a renilla luciferase expression construct and the indicated amounts of antisense LNA or LNAzyme, and the HIV-1 production was measured by CA-p24 ELISA 72 hours later. (A) HIV-1 production in the presence 20 nM of the four different HIV-1 specific LNAs and 2 LNA controls (Mock 1 and Mock 2). (B) Measuring HIV-1 production in the presence of low range concentration of LNA_PBSD _(0.16–20 nM). The inhibition is calculated as the average value of two independent experiments where the relative CA-p24 expression is normalized for unspecific inhibition of renilla expression. (C) Comparing the inhibitory capacity of DNA versus LNA containing antisense or 10–23 enzymes targeted to the DIS and PBSD regions. The identity and the concentration of the oligonucleotide are indicated below. The assay was performed in duplicates.

To evaluate the capacity of the antisense LNA to inhibit cellular HIV-1 expression the expression of the viral Gag derived CA-p24 protein was measured in the presence of 20 nM of the four different antisense and two mock LNAs (Fig. 5A). HEK 293-T cells were co-transfected with HIV-1 LAI genomic DNA plasmid, renilla luciferase plasmid and the LNAs. The CA-p24 production was strongly affected by all the HIV-1 specific LNAs, particularly by LNA_PBSD _and LNA_AUG_, which reduced protein production by 22- and 12-fold, respectively (Fig. 5A). In contrast, the internal luciferase control was only marginally affected (+/- 2-fold) by some of the LNAs (data no shown). The effect of the most potent LNA_PBSD _construct was investigated further at lower concentrations (Fig. 5B). Notably, CA-p24 expression was severely affected at concentrations as low as 4 nM (15-fold inhibition) and a complete block was apparent at 20 nM LNA_PBS _(Fig. 5B). This block was specific for HIV protein expression since the renilla luciferase signal was not affected at these concentrations of LNA (data no shown).

To directly compare the inhibitory potential of the different strategies, different concentrations of oligo constructs (asDNA, asLNA, DNAzymes and LNAzymes) targeted to the PBS and DIS targets were tested for their ability to inhibit CA-p24 production in HIV-1 transfected cells (Fig. 5C). LNA_PBS _and LNA_DIS _were clearly the most potent inhibitors, leading to almost complete knock down (below detection) at 20–100 nM and to a 3- and 18-fold inhibition, respectively, at 4 nM. In contrast, the DNA antisense oligos showed little effect. Notably, both the DNA- and LNAzymes led to a specific knock down. The LNAzymes were more effective than DNAzymes, giving a 100-fold knockdown at 100 nM (Fig. 5C). However, the LNAzymes also exhibited significant cell toxicity when applied at 100 nM concentration (data no shown).

## Discussion

A major concern in the design of therapeutic antisense strategies against highly structured viral RNA genomes is the inaccessibility of the target sequence. To overcome this barrier we have chosen 4 targets in the HIV-1 genome that were previously selected as optimal annealing sites in vitro, and we tested them as targets for DNA and LNA antisense oligonucleotides, and DNA- and LNA-zymes. The antisense oligonucleotides were tested *in vitro *for their ability to interfere with reverse transcription and RNA dimerization and all inhibitors were assayed *in vivo *for their capacity to inhibit HIV-1 production in a cell culture assay.

Reverse transcription of viral RNA into double stranded DNA is an essential step in the retroviral replication cycle. A comparison of the antisense oligos for their ability to block this reaction revealed that all LNAs, except for LNA_DIS_, caused a near complete block in reverse transcription. In addition to a significant level of read through, two pause sites were observed for LNA_DIS_: one site mapped to the expected 5'end of the LNA, the other corresponded to the 3' nucleotide of the DIS loop (Fig. 1B). A likely interpretation for this observation is that a significant part of the LNA_DIS _molecules anneal only to the exposed loop region, yet is unable to unzip the DIS stem in the HIV-1 RNA. This may explain the partial effect on reverse transcription. In contrast, RNA dimerization is nearly 100% blocked by LNA_DIS_, suggesting that annealing of the LNA antisense to the single stranded loop of the DIS hairpin is very effective and sufficient to block dimerization.

The inhibitory potential of the antisense oligos in vivo was dramatically improved upon LNA incorporation. Especially the LNAs targeted to the PBSD, DIS and AUG regions were strong inhibitors of CA-p24 capsid protein expression. The advantage of LNA may in part rely on higher stability in the cells, but increased stability of the interaction between LNA and target most likely also plays an important role. The mechanism for the observed inhibition may involve numerous steps in the viral life cycle. All of the LNA gap-mers may degrade the mRNA via an RNase H dependent pathway and, if not degraded, block scanning of the ribosome during cap-dependent translation or the HIV-1 reverse transcriptase while copying the RNA template. In addition, the individual LNA may also have more specific actions that cannot directly be assessed in our single-round HIV-1 expression assay: the LNA_PBSD _may interfere with tRNA binding to the PBS and subsequent initiation of reverse transcription and the LNA_DIS _may, in addition to dimerization, hinder effective packaging of the genome into viral particles. The LNA_SD _covers the major splice donor site and may therefore interfere with splice site recognition but RNA packaging may also be disturbed based on the contribution of the region to this process [32-35]. Finally, the LNA_AUG _that covers the Gag initiation codon may interfere with the assembly of translational initiation complexes or disturb the long distance interaction recently reported between this region and upstream sequences [36,37]. Hence, the multi-functional capacities of the LNAs applied in this study may be beneficial for their antiviral effect.

Of the four antisense targets we tested, LNA_AUG _was particular interesting since it overlaps with the previously described phosphorothioate modified 25-mer antisense oligonucleotide, GEM91 [38-40]. In cell culture experiments this oligo has been shown to inhibit HIV-1 replication for up to 20 days when applied at 1 μM [38,40] and pharmacokinitical studies have been initiated in HIV-1 patients but abounded due to dose-limiting toxicity [41]. Considering that the general improved affect of LNAs compared to DNA it will be interesting to further develop the AUG directed LNA oligos as antiviral drugs for clinical use. In another study several antisense polyamide nucleotide analog (PNA) oligonucleotides targeted to the TAR stem-loop were tested, and one of the PNAs was found to have significant inhibitory potential on HIV-1 protein expression [42], however only at concentrations of more than 1 mM [43]. The efficacy of these constructs were recently improved by conjugating cell penetrating peptides to them [44].

In general the TAR-tat interaction have been objective for several antisense approaches using either LNA modified oligos [45,46], LNA/DNA aptamers [47], mixmer of 2'-OMe and LNA modified oligos [48,49] or PNA modified oligos [50-53]. These results do indeed indicate that the TAR region is a useful antisense target site and that various antisense oligos can inhibit the replication of HIV in different cell systems. The absence of binding sites in the TAR region in our screen suggest that this region is less accessible then the sites we have selected.

In another report, antisense gap-mer LNAs targeted to the DIS region have been tested [54]. One of these oligos resembles our LNA_DIS_, but it is shifted a few nucleotides upstream and is two nucleotides shorter. A relatively modest 2-fold inhibitory effect was described, both in terms of in vitro dimerization and on HIV-1 expression in vivo in the presence of 160 nM oligonucleotide. This implies that small changes in target selection may have a dramatic effect, which is consistent with our in vitro binding studies [22]. However the results are not directly comparable since Elmén et al. used a subtype A HIV-1 strain that exhibits a different DIS loop sequence than the subtype B used in this report. The strong dependence on target availability creates a risk that adaptive mutations in the HIV-1 genome will render the antisense oligo less effective. It may therefore be favourable to combine the most effective LNA in future tests.

As an alternative to the antisense technology we tested the inhibitory capacity of one of the best-characterized DNAzymes, "10–23". The cleavage efficiency of this enzyme has previously been reported to be highly variable, which has limited its use [12,31,55-57]. The reason for this is believed to be the poor annealing of the DNAzymes to their targets. In this report, DNAzymes were targeted to the PBSD and DIS sites. The PBSD site was selected because it represents the most efficient target site for antisense molecules and hence may also be a good target for a DNAzyme and the DIS target due to it is partially inaccessible to at least one of the arms in the DNAzyme, allowing us to test the hypothesis that incorporation of LNA residues can enhance the cleavage efficiency ([30]; Fig. 1B). Indeed, we also observed that the activity of the DNAzyme directed towards the DIS target was strongly induced upon LNA incorporation, whereas the PBSD specific DNAzyme was approximately equally active in its modified and unmodified form. The LNAzyme_DIS _abrogated CA-p24 expression compared to the unmodified DNAzyme by at least 10-fold at 100 nM, which is consistent with a previous study on a cellular mRNA target [58]. In light of our *in vitro *data, this induction is most likely a result of increased cleavage of the target rather then being a stability issue. This interpretation is consistent with the much more modest effect observed when introducing LNA modifications in the more active DNAzyme_PBSD_, both *in vitro *and *in vivo*. A potential disadvantage from introducing LNA residues in the arms of a DNAzyme is that the interaction between the LNAzyme and the target becomes too strong, which may reduce the turned over. This may explain why we never reached the point of multiple turnover using LNA modified enzymes, indicated by nearly complete digestion of target at sub-stoichiometric concentration of the enzyme.

As for antisense constructs, DNAzymes targeted against randomly selected sites are generally inactive. For instance, out of 8 DNAzymes targeting the HIV-1 TAR region, only 2 yielded detectable cleavage products and a relatively high concentration (1 μM) of inhibitor yielded only a 5–10 fold reduction in CA-p24 expression [56]. Both the pre-selected target sites tested here were cleaved by the DNAzymes reducing CA-p24 expression at a 10-fold lower concentration. The difficulties in rationally predicting efficient targets for nucleotide enzymes is also reflected by the observation that a DNAzyme directed against the natural tRNA primer binding site, which is generally assumed to be available for annealing, is unable to cleave the HIV-1 RNA (M.R.J. and J.K., unpublished observations).

siRNA targeted to the four highly accessible regions in the 5'UTR had almost no effect on HIV-1 gene expression (J.H., M.R.J. and J.K. unpublished observation). However, a direct comparison with the antisense approach is not meaningful since the selected target sequences are suboptimal using state of art design rules for siRNA [59]. Efficient knock down of HIV-1 expression by RNAi has been demonstrated using other targets [21,60-62] and this approach is generally considered to be more potent than antisense. However, we find that the inhibitory effects observed with selected LNA antisense constructs in the low nanomolar range is able to match some of the best HIV-1 specific siRNAs reported in literature [63].

## Conclusion

Four sites that were pre-selected as highly accessible regions in the HIV-1 leader were accessed as potential targets for various antisense based technologies and we conclude that antisense LNA targeted to specific sites in the PBS and the DIS regions were the most effective inhibitor of HIV-1 expression. LNA may have additional advantages for in vivo applications, such as more efficient cell uptake and increased stability. Moreover, the lower molecular weight and single stranded nature of LNA makes it potentially more inexpensive to synthesise in large quantities than double stranded siRNA. LNA therefore provides a serious alternative platform for development of therapeutics for human diseases.

## Methods

### Constructs

The plasmids pUC18-LAI and pUC18-LAI-1–444 contain +1–355 nucleotides and +1–444, respectively, of the HIV-1 genome sequence of the LAI isolate behind a T7 promoter and have been described earlier [64]. LNA oligonucleotides (Exiqon) were all designed as gap-mers with 5 LNA residues flanking a 10-mer phosphorothioate modified DNA body, except from MOCK-2 that were an 18-mer with only 4 LNA residues at each termini. Both MOCK-1 and MOCK-2 contain random sequences without extensive match to human or HIV-1 sequences. The LNAzymes (Exiqon) and DNAzymes (DNA Technology) were constructed as "10–23" enzymes [12] with an arm length of 9 nucleotides. RNA transcription was performed as described earlier [22].

### Primer extension assay

The primer extension assay was performed using 1 pmol RNA spanning the first 444 nucleotides of the HIV-1 genome and 80 fmol 5'end-labeled RT primer (5'-CCTTAACCGAATTTTTTCCC-3') complementary to position 384–401. The template and the primer were annealed for 2 min at 90°C in a total volume of 6 μl annealing buffer (100 mM Tris-HCl, pH 7.5, 400 mM KCl) followed by 5 min at room temperature. Then 1 pmol LNA oligonucleotides were added and incubated for 20 min at 50°C. Reverse transcription and gel analysis was performed according to Damgaard et al. [65,66].

### Dimerization assay

One pmol [γ-P^32^] HIV-1 leader RNA and five pmol LNA oligonucleotide was incubated in 20 ul of water at 85°C for 5 min and then snap-cooled on ice for 5 minutes. The buffer was adjusted to dimerization conditions (50 mM Na-cacodylate; pH 7.5, 250 mM KCl, 5 mM MgCl_2_) and incubated at 37°C for 30 min in The sample was analyzed on a 6% native TBM gel (50 mM Tris-borate; pH 8.3, 5 mM MgCl2) and autoradiographed using phosphor image screens (Biorad).

In the kinetic assay, 1 pmol HIV-1 leader RNA was allowed to predimerize for 30 min at 37°C at dimerization conditions and then either 5 pmol LNA, DNA or RNA DIS oligonucleotide was added. After 0, 30, 120 and 240 minutes aliques were taken out and placed on ice before analyzed on a 6% native TBM gel and autoradiographed on phosphor image screens.

### DNazyme and LNazyme cleavage assay

One hundred nmol 5' end labelled leader (+1–355) RNA was incubated at 85°C in 7 μl water for 5 min and cooled on ice for 5 min. The RNA were then mixed with 5 nmol, 100 nmol or 2 pmol DNAzymes or LNAzymes and incubated at 37°C for 1, 2, 4 or 24 hours in DNazyme buffer (10 mM MgCl_2_, 50 mM Tris-HCl; pH 8.0) and stopped with 100 mM EDTA. The samples were precipitated and analyzed on an 8% denaturing polyacrylamide gel run at 18 W and analyzed by phosphor imaging (Biorad).

### HIV-1 production assay

HEK 293-T cells were seeded one day before transfection at 150.000 cells/ml/well in a 24-well plate. Transfection was performed at 40% confluency in duplicate using Lipofectamine-2000 (Invitrogen) in 400 μl medium without antibiotics. Per transfection, 100 ng of HIV-1 genomic LAI plasmid was diluted in 50 μl OPTIMEM and the respectively final concentration of the various oligonucleotides. Two μg Lipofectamine was added to 48 μl OPTIMEM and incubated for 5 min at RT. The diluted DNA and lipofectamine were combined to a final sample volume of 100 μl. This mixture was incubated for 20 min at 20°C before adding to cells. Six hours post-transfection 1 ml medium containing antibiotics replaced the original medium. Three days post-transfection 100 μl from the culture media was collected and inactivated by adding 10 μl 0.1% Empigen (final concentration) and heating at 65°C for 30 min. Production of HIV-1 CA-p24 was measured with p24 enzyme-linked immunosorbent assay (ELISA). As an internal control, 2.5 ng pRL was included and the Renilla luciferase expression levels were measured using the Dual-luciferase Reporter Assay System (Promega).

## Abbreviations

HIV: Human Immunodeficiency Virus

LNA: Locked Nucleic Acid

5'-UTR: 5' Untranslated Region

RT: Reverse Transcriptase

TAR: Trans-activation response element

PBS: Primer binding site

DIS: Dimer initiation site

SD: Major splice donor

PSI: Packaging signal

PBSD: Primer binding site downstream

## Competing interests

The author(s) declare that they have no competing interests.

## Authors' contributions

MRJ: Construct design, primer extension assay, enzymatic cleavage assay and manuscript preparation

JH: HIV-1 production assay

JW: LNA oligonucleotide and LNAzyme design

BB: Manuscript preparation

JK: Experimental design, manuscript preparation

All authors read and approved the final manuscript.
